# One Year Follow-Up Assessment of Impact of Rigorous Diet Regimen and Adequate C-PAP Therapy on Obese Patients with Obstructive Sleep Apnea Syndrome: A Retrospective Study

**DOI:** 10.3390/jcm13216360

**Published:** 2024-10-24

**Authors:** Pierluigi Carratù, Silvano Dragonieri, Vitaliano Nicola Quaranta, Onofrio Resta, Piero Portincasa, Vincenzo Ostilio Palmieri, Giovanna Elisiana Carpagnano

**Affiliations:** 1 Institute of Internal Medicine “A. Murri”, Department DIMEPRE-J, University of Medicine, 70124 Bari, Italy; piero.portincasa@uniba.it (P.P.); vincenzo.palmieri@uniba.it (V.O.P.); 2 Institute of Respiratory Diseases, Department DIBRAIN, University of Medicine, 70124 Bari, Italy; silvano.dragonieri@uniba.it (S.D.); vitalianonicola.40@gmail.com (V.N.Q.); onofrio.resta@uniba.it (O.R.); giovannaelisiana.carpagnano@uniba.it (G.E.C.)

**Keywords:** obesity, OSAS, C-PAP, diet, BMI, AHI

## Abstract

**Background/Objectives:** This study evaluated the impact of continuous positive airway pressure (C-PAP) therapy combined with a rigorous diet regimen on obese patients with obstructive sleep apnea syndrome (OSAS). **Methods:** Sixty obese patients (BMI ≥ 30) diagnosed with severe OSAS were recruited in order to establish the evaluation of CPAP therapy with different extents of adherence to a rigorous diet regimen. After one year, significant improvements were observed. **Results:** BMI reduced by 12.32%, apnea–hypopnea index (AHI) by 22.04%, oxygen desaturation index (ODI) by 15.87%, total sleep time with oxygen saturation below 90% (TST90%) by 25.2%, and Epworth Sleepiness Scale (ESS) scores by 21.74%. Patients were, then, divided into three groups, based on adherence to the restricted diet, as well as to the correct use of the nocturnal C-PAP, showing different reductions in BMI, AHI, ODI, TST90%, and ESS, according to their adherence, based on the sum of % reduction in BMI + AHI into three groups. **Conclusions:** These findings underscore the effectiveness of combining C-PAP therapy with a strict diet in improving OSAS symptoms and overall health in obese patients. Future studies with larger cohorts and longer follow-up periods are needed to confirm these results and explore the long-term benefits of this integrated approach.

## 1. Introduction

The Obstructive Sleep Apnea Syndrome (OSAS) is defined as a clinical condition characterized by repetitive episodes of partial or complete airway obstructions that, occurring during sleep, induce oxyhemoglobin desaturation and sleep fragmentation [1]. In the OSAS, the upper airway collapse is caused by inadequate muscle tone of the tongue and/or airway dilator muscles [2], and the subsequent, frequently repeated, breathing gaps lead to interruption of the regularity of the sleep cycle phases [3], which, in turn, involves several clinical manifestations such as increased daytime sleepiness, morning headaches and cognitive decline [4]. This pathological condition is also strongly associated with serious health complications, especially with increased cardiovascular risks [5], such as hypertension, atrial fibrillation, heart failure, stroke, and metabolic disorders, glucose intolerance, hyperinsulinemia, type 2 diabetes, and Alzheimer disease [6,7,8,9].

Moreover, data from epidemiological studies showed that although OSAS is largely underestimated, it is a very common condition, and it has been established that its prevalence both in developing and developed countries is 3–7% in men and 2–5% in women [10,11,12,13]. From an etiopathogenetic point of view, the risk of developing this syndrome has been associated with several risk factors (e.g., age older than 50, male gender, family history of OSAS, tobacco and alcohol consumption, obesity, hypertension, congestive heart failure, atrial fibrillation, atherosclerosis and endothelial dysfunction, retrognathia, acromegaly, type 2 diabetes, chronic nasal congestion, asthma) that are widely accepted worldwide [14,15].

Among the risk factors, obesity is considered the most important one, which is involved in the onset of OSAS [16]. Indeed, interestingly, the presence of sleep apnea has been detected in about 40% of overweight subjects, and its prevalence rises significantly in severe obese people. On the other hand, it has been observed that about 50% of OSAS patients are obese [17]. The etiopathogenetic mechanism behind this close association between obesity and OSAS has been attributed to an increased fat deposition in specific anatomical sites, such as tissues surrounding the upper airway and around the chest by facilitating the collapsibility of the upper airway and reducing the chest compliance, respectively, inducing a significant worsening of OSAS symptoms [18,19]. Currently, the results of several recent studies demonstrated that the relationship between obesity and OSAS is extremely complex, and it involves different mechanisms of action (e.g., vascular endothelial dysfunction, metabolic dysregulation, inflammation, oxidative stress, recurrent hypoxia, enhanced sympathetic nerve activity), suggesting that OSAS and obesity might be interrelated with both conditions, increasing the adverse effects of the other [16,20,21].

Regarding to the currently available therapeutic options for treating this syndrome, continuous positive airway pressure (C-PAP) via nasal or oro-nasal mask is considered the gold standard since it is very effective in improving airway patency during sleep [2,22,23]. However, in some cases, patient compliance with the use of C-PAP can be limited, thus determining an inadequate adherence to the therapeutic protocol or a discontinuous treatment [24,25]. In this context, regardless of the treatment approach, but considering the significant interplay between OSAS and obesity, an important counselling activity concerning the importance of losing weight should be recommended to all OSAS patients in conditions of overweight or obesity. Usually, this clinical recommendation on weight loss translates into practice by following healthy lifestyles (regular physical activity and/or a healthy and balanced diet) [26]. However, the healthy lifestyle is rarely achieved by OSAS patients.

In the present study, we retrospectively evaluated the combined impact of continuous positive airway pressure (CPAP) therapy and adherence to a rigorous diet regimen on clinical outcomes in obese (BMI ≥ 30) patients with OSAS. Specifically, we assessed changes in BMI, AHI, and other key indicators of OSAS severity after one year of treatment. To further analyze the impact, patients were divided into three groups based on their level of adherence to both CPAP and the diet regimen. This division allowed us to compare clinical outcomes across different adherence levels and highlight the effectiveness of this integrated therapeutic approach.

## 2. Materials and Methods

### 2.1. Subjects

In this retrospective study, we recruited 60 obese individuals (22 females, mean age 56.75 ± 14.90, range 31–90) who were admitted to the ambulatory of Sleep Disorders of the Pulmonary Clinical of University of Bari during the period from March 2017 to March 2018 with a well-documented diagnosis of OSA (Table 1). All patients who were smokers were excluded from the study, as well as all the patients with systemic diseases, which constituted contraindication to the dietary regimen or to the CPAP therapy. Patients were grouped based on their adherence to CPAP therapy and diet regimen in order to evaluate how varying adherence levels impacted clinical outcomes, but the primary focus of the study remained on assessing the overall effectiveness of combining CPAP therapy with a strict diet regimen in managing OSAS symptoms.

Initially, potential participants were screened carrying out a medical examination to collect the main sociodemographic data, symptoms, occupational history, and an accurate physical examination with particular reference to the respiratory tract and ear, nose, and throat system. Furthermore, weight and length were measured to calculate the correct BMI. Importantly, in order to have a clinical picture that was as exhaustive as possible, in the anamnestic collection, particular attention was paid to the analysis of the therapeutic protocols that participants were already following to manage the OSAS and to the evaluation of the previously undertaken lifestyle weight loss interventions (e.g., diet, physical exercise, behavioral programs or any combination of them). Moreover, adherence to the diet regimen and CPAP therapy was assessed through self-reported data provided by participants during their follow-up visits at the end of the one-year study period. Participants were asked to retrospectively report their adherence to the diet and their compliance with the CPAP therapy. To mitigate recall bias, participants were guided through structured interviews, where they were asked specific questions about the frequency and consistency of their diet and therapy adherence over the past year.

We acknowledge the limitations inherent in self-reporting, particularly in retrospective studies, and have taken steps to improve accuracy by cross-referencing patient responses with medical records, where available, such as weight and BMI changes at different follow-up points.

No specific medications for weight loss were prescribed during the study period. The study focused on the natural effect of diet and CPAP adherence without pharmacological intervention. Participants were advised to maintain physical activity levels, although these were not systematically tracked during the study, and no formal exercise intervention was part of the protocol. All patients were adequately informed regarding the purposes of the study, and they were asked to sign a written informed consent form. This study has been performed according to the Declaration of Helsinki, and it was approved by our local ethical Committee (Number of protocol 44696/DS 06-03-2009).

### 2.2. Study Design

The study retrospectively assessed the impact of different levels of diet and C-PAP adherence on various clinical outcomes in obese patients with OSAS. The patients were divided into three groups based on their level of diet and CPAP adherence: Perfect Diet and CPAP Adherence, Moderate Diet and CPAP Adherence, and Insufficient Diet and CPAP Adherence, measured as the sum of reduction in BMI + AHI, after 1 year of follow-up.

### 2.3. Body Mass Index

Height and weight data were collected during the first medical examination and then after one year at the time of the follow-up. This information was used to calculate the BMI, which is a simple, widely used and helpful tool for classifying underweight, overweight and obesity status in adults. According to the BMI grading system of the World Health Organization, participants were categorized as underweight (<18.5 kg/m^2^), normal weight (18.5–24.9 kg/m^2^), pre-obese (25–29.9 kg/m^2^), class I or mild obese (30–34.9 kg/m^2^), class II or moderate obese (35–39.9 kg/m^2^) and class III or severe obese (≥40 kg/m^2^). The mean of the BMI of the recruited individuals was 37.9 ± 3.29, and the range was 31–48. Overall, 11.7% of patients were classified as class I or mild obese (mean 32.6 ± 0.98), 61.7% of participants were grouped as class II (mean 37.1 ± 1.33), 26.6% of OSAS were classified as class III (mean 42.06 ± 2.14).

### 2.4. Polysomnography

Polysomnography (PSG) is the gold standard for the diagnosis of OSAS, and it is fundamental both to evaluate its severity as well as to identify the best therapeutic choices. This non-invasive diagnostic test is performed in a sleep laboratory under technician supervision, and it consists of continuously monitoring nocturnal sleep by recording several cardiac, neurological, and respiratory parameters. Therefore, the use of PSG allows ascertaining and quantifying the occurrence of respiratory airflow cessation episodes, the presence of loud intermittent snoring, the detection of nocturnal awakening, and the possible association with oxygen desaturation. In this regard, the apnea–hypopnea index (AHI) is the ratio between the total number of apnea and apnea, plus hypopnea episodes and the total sleep duration expressed in hours. The severity of OSAS is defined as mild, moderate or severe when the AHI is in the range of 5–15/h, 15–30/h or ˃30/h, respectively [27].

All patients recruited in the study underwent PSG using a digital multichannel polygraph Vital-Night (Vital Aire, Milan, Italy) at Time 0 (T0). During the full-night sleep study, we carried out a comprehensive collection of several parameters such as the total number of apneic and hypopneic episodes, the number of central and obstructive apneas, the AHI, the oxygen desaturation index (ODI), and the total sleep time (TST) with oxygen saturation lower than 90% (TST 90%). According to the American Academy of Sleep Medicine criteria, an apnea was identified as cessation of airflow for at least 10 s with continued effort (obstructive) or lack of effort (central) to breathe, whereas hypopnea has been defined as >50% decrease in a valid measure of airflow without an association with oxygen desaturation or arousal, or alternatively, as a lower airflow reduction in association with oxygen desaturation of >3%, or an arousal for at least 10 s [28]. The second PSG, conducted at the 12-month follow-up, was performed without the use of CPAP to assess the natural progression of OSA symptoms and the impact of weight loss without the influence of the CPAP therapy.

### 2.5. Epworth Sleepiness Scale Questionnaire

This is a validated tool to assess the excessive daytime sleepiness (EDS). The Epworth Sleepiness Scale (ESS) is a self-administered questionnaire that is based on eight items that refer to different type of situations, some of which are well known for being particularly soporific [29]. Participants can answer each question by assigning a score on a 4-point scale, ranging from 0 (would never fall asleep) to 3 (high chance of dozing), that indicates the frequency with which drowsiness might occur during the occupations described in the items. The scores assigned to each question are then added together to provide the total ESS score (0–24) indicating the subject’s degree of sleepiness [30]. ESS scores higher than 9 are suggestive of a high level of daytime sleepiness. The ESS questionnaire was self-administered during the first medical examination, and then one year after, although it has been shown to be more appropriate if administered by physicians [31].

### 2.6. Diet Regimen

The diet regimen followed in this study was the Montignac Diet, which is based on the glycemic index (GI) of foods [32]. The Montignac Diet emphasizes the consumption of low-GI carbohydrates to regulate blood sugar levels and promote weight loss. Foods with a low GI release glucose slowly into the bloodstream, preventing spikes in insulin levels, which can lead to fat storage. The diet includes a high intake of fiber-rich vegetables, lean proteins, and healthy fats while avoiding high-GI foods such as refined sugars and processed carbohydrates. This approach not only aids in weight loss but also helps in managing insulin resistance and improving overall metabolic health [32].

### 2.7. Statistical Analysis

The results are presented as mean ± SD. The paired Student’s *t* test was used to compare the following parameters: BMI, PSG indexes, and ESS test at Time 0 and after 1 year follow-up, T1. The Pearson’s r correlation test was performed to calculate the strength of the linear relationship between two variables. The ANOVA test was used to evaluate statistical differences among the three groups calculated at T1. A *p* value ≤ 0.05 was considered statistically significant. Statistical analyses were performed using NCSS10 Statistical Software, version 22 (NCSS, LLC, Kaysville, UT, USA).

## 3. Results

### 3.1. Age and Sex Distribution

The mean age of participants was similar across the three groups, and no significant differences were found between them (*p* = 0.515). Similarly, there were no significant differences in the sex distribution between the groups (*p* = 0.241) (Table 1).

### 3.2. Body Mass Index (BMI)

At the beginning of the study (T0), there was a statistically significant difference in BMI among the groups (*p* = 0.010) with the Perfect Diet and C-PAP Adherence group having a higher baseline BMI compared to the Moderate Diet and C-PAP Adherence group as well as Insufficient Diet and C-PAP Adherence groups. After the intervention (T1), the BMI significantly decreased in all groups with the most substantial reduction observed in the Perfect Diet Adherence group (*p* = 0.000). This suggests that stricter adherence to the diet had a more pronounced effect on weight reduction (Table 2, Table 3 and Table 4).

### 3.3. Apnea–Hypopnea Index (AHI)

**Figure 1:**
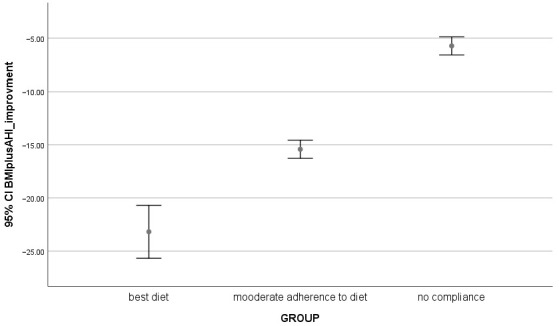
Comparison among groups, 95% CI of BMI + AHI negative improvement into three groups of patients.

AHI, a key measure of OSA severity, was also significantly different between the groups at baseline (T0) (*p* = 0.000). The Moderate Diet and C-PAP Adherence group had the lowest AHI at baseline, while the Perfect Diet and C-PAP Adherence group showed the most severe apnea. However, after the intervention (T1), all groups showed significant improvements in their AHI scores with the Perfect Diet and C-PAP Adherence group experiencing the most marked reduction (*p* = 0.000) (Figure 1, Table 2, Table 3 and Table 4).

### 3.4. Time Spent with Oxygen Saturation Below 90% (TST90)

TST90, which reflects the time spent in a hypoxic state during sleep, was significantly different across the groups at baseline (*p* = 0.004). Following the intervention, all groups experienced a reduction in TST90 with the Perfect Diet and C-PAP Adherence group showing the greatest improvement (*p* = 0.000). This indicates that rigorous diet together with C-PAP adherence may contribute to better oxygenation during sleep in OSA patients.

### 3.5. Epworth Sleepiness Scale (ESS)

The ESS scores, which measure daytime sleepiness, did not show significant differences between the groups at baseline. However, at the end of the intervention, there were significant improvements in the ESS scores particularly in the Perfect Diet and C-PAP Adherence group (*p* = 0.000). Patients in this group reported feeling less sleepy during the day, which suggests that better diet and C-PAP adherence may lead to improvements in overall sleep quality and daytime functioning) (Table 2, Table 3 and Table 4).

### 3.6. Within-Group Comparisons

Within each group, there were significant improvements from baseline to the end of the study in terms of both BMI and AHI. These changes were most pronounced in the Perfect Diet and C-PAP Adherence group, where patients experienced the greatest reductions in BMI and AHI (*p* < 0.05). This suggests that strict adherence to the diet and C-PAP had a more substantial impact on decreasing weight and OSA severity as compared to moderate or insufficient adherence (Table 5).

## 4. Discussion

It is widely recognized that an adequate treatment with C-PAP represents the gold standard for the OSAS therapy [23], reducing the respiratory and cardiovascular failures as well as the mortality of these patients [33]. On the other hand, a restricted dietary regimen has also demonstrated that the behavioral medicine intervention facilitated changes in eating behavior in patients with OSAS and overweight [34]. No data are available in the literature about a large cohort study of obese patients with OSAS after 1 year follow-up of restricted dietary regimen together with a constant and adequate C-PAP treatment.

Our study demonstrated that a rigorous dietary regimen, together with the systematic application of C-PAP, for at least 8 h per night, was effective in reducing the clinical severity and the diurnal symptoms of OSAS among a cohort of obese individuals. We observed, for all the different parameters investigated, an important and statistically significant decrease in their mean levels compared to the values obtained at T_0_. Importantly, the average levels of sleep apneas and the number of AHIs have undergone a drastic decrease in all 60 patients with significant statistically differences among the three groups (Table 2).

Although obesity is the main risk factor for the development of OSAS, currently, it is widely appreciated and accepted that the pathogenesis of this syndrome is multifactorial, involving different organs and systems such as lung, adipose tissue, and gut microbiota and, especially in obese patients, it is not simply (or only) dependent on a pure mechanical load acting on the upper respiratory tract. Therefore, it is plausible to hypothesize that targeted intervention against a single risk factor (i.e., obesity) can be curative only in a limited number of patients with OSAS. Another important aspect is represented by the high attention paid by some patients to accurately use the nocturnal C-PAP, which is evidenced by a consistent reduction in apnea/hypopnea (AHI) events. We found a significant reduction in ESS score, thus demonstrating greater reactivity and less daytime sleepiness. When the patients were divided into three groups, this evidence was much more extended, demonstrating a terrific difference of these results. In the group of low adherence to the restricted dietary regimen, together with a not consistent use of C-PAP for at least 8 h per night, the 1-year follow-up showed a reduction of 2.62% of BMI, 8.6% of AHI, 8.32% of ODI, 9.6% of TST90% and 10.1% of ESS. In the group that obtained medium results after 1 year follow-up, we observed a reduction of 15% of BMI, 26.4% of AHI, 18.7% of ODI, 31% of TST90%, and finally 27.3% of ESS. In group 3, who demonstrated the best performances after 1 year of follow-up, we found a reduction of 17.8% of BMI, 30% of AHI, 25.8% of ODI, 35.5% of TST90% and 29% of ESS, demonstrating an accurate and systematic observation of the recommendations of physicians with some improving, which considerably ameliorate their quality of life. Table 2 illustrates the comparison among the three groups.

Our approach, emphasizing rigorous diet and adequate use of nocturnal CPAP, aligns with current recommendations for the management of obesity and OSAS [35,36,37], where lifestyle modification stands as the cornerstone of treatment. It is also more accessible and potentially less costly than other therapy (for example bariatric surgery), presenting fewer risks and complications. Given the chronic nature of obesity and OSAS, sustainable lifestyle changes offer a long-term solution that can be adjusted and maintained over time to ensure continuous health benefits. Although several studies have explored the independent effects of CPAP therapy and dietary interventions in OSAS [38,39], there remains a relative lack of large-scale, long-term investigations into the combined impact of these interventions, particularly with a focus on strict dietary adherence over extended follow-up periods. Our study aims to contribute to this growing body of research by providing a one-year follow-up and highlighting the relationship between adherence to both interventions and OSAS outcomes.

Despite the promising findings, this study has several limitations:

First, the sample size of 60 participants is relatively small, limiting the generalizability of the results. Future studies with larger cohorts are needed to confirm these findings and provide more robust data. Second, this study is a retrospective observation, which can introduce various biases, such as selection bias and recall bias. Prospective, randomized controlled trials are necessary to establish causality and eliminate these biases. Third, the follow-up period of one year, although sufficient to observe significant changes, may not be adequate to assess the long-term sustainability of the observed benefits. Longer follow-up periods are required to evaluate the enduring effects of the combined CPAP therapy and dietary regimen on OSA and obesity. Fourth, the adherence to the dietary regimen and CPAP therapy was self-reported, which can lead to an overestimation or underestimation of adherence levels. The sum of the reduction in the two most important markers of disease, such as BMI and AHI, is due to a personal choice and not to a standard commitment. Objective measures of adherence, such as device usage data for CPAP and dietary logs or biochemical markers for diet compliance, would provide more accurate assessments. Fifth, in this study, we observed a significant reduction in AHI after one year of combined CPAP therapy and a strict dietary regimen. However, it is important to acknowledge that the reduction in AHI may reflect a combination of both weight loss and a residual effect of continuous C-PAP usage. The current literature suggests that AHI scores can be lower after the first night without CPAP with values potentially rising over the course of subsequent nights. Thus, distinguishing between the impacts of weight loss and any residual CPAP effect on AHI in our study is challenging. Future studies should consider implementing a washout period or performing multiple PSG sessions without CPAP to better isolate the specific contributions of weight loss to AHI reduction. Another limitation of our study is the baseline difference in BMI between the groups. Since BMI is an important factor in the evaluation of OSAS severity, these differences could have influenced the outcomes of the study. Although our analysis was designed to assess the impact of both CPAP and diet adherence on clinical outcomes, future studies should consider a more careful matching of baseline BMI across groups or applying statistical adjustments to account for these variations.

Lastly, the study was conducted at a single center, which may limit the applicability of the results to other populations or settings. Multicenter studies are needed to validate these findings across several groups.

## 5. Conclusions

In conclusion, our findings advocate for the integration of a structured weight loss program involving diet and correct adherence to CPAP into the treatment plans for patients with OSAS. Such programs not only reduce the symptoms of OSAS but also contribute to the overall improvement of patients health and quality of life, reinforcing the need for multidisciplinary approach to manage this complex condition effectively.

These preliminary results may also be confirmed by a significative increase in average years of life, thus reducing the mortality, by conducting a prospective study after 10 years of follow-up.

## Figures and Tables

**Table 1:** Entire population, anthropometric and PSG parameters.

60 Individuals Obese, with OSA	
AGE m ± sd	56.75 ± 14.90
SEX f n (%)	20 (33.3)
BMI m ± sd	38.10 ± 3.52
AHI m ± sd	46.20 ± 13.13
ODI m ± sd	45.04 ± 14.10
TST90 m ± sd	25.45 ± 8.34
ESS m ± sd	14.35 ± 2.79

**Table 2:** Comparison among groups.

	Group 1 Perfect Diet and CPAP Adherence Compliance N = 20	Group 2 Moderate Diet and CPAP Adherence Compliance N = 20	Group 3 Insufficient Diet and CPAP Adherence Compliance N = 20	*p* Value
AGE m ± SD	54.22 ± 16.88	59.66 ± 13.25	56.37 ± 14.60	0.515
SEX n (%)	4 (20)	9 (45)	7 (35)	0.241
T0_BMI m ± SD	39.75 ± 3.99	36.45 ± 2.23	38.10 ± 3.44	0.010
T0_AHI m ± SD	53.81 ± 13.99	37.76 ± 5.72	47.02 ± 13.06	0.000
T0_ODI m ± SD	50.84 ± 15.70	41.19 ± 12.29	43.08 ± 12.83	0.070
T0_TST90 m ± SD	28.20 ± 9.12	20.55 ± 5.16	27.60 ± 8.24	0.004
T0_ESS m ± SD	14.85 ± 3.39	13.95 ± 1.93	14.25 ± 2.91	0.591
T1_BMI m ± SD	32.70 ± 3.35	31.00 ± 2.44	36.40 ± 3.21	0.000
T1_AHI m ± SD	37.68 ± 11.88	27.80 ± 5.59	42.98 ± 11.88	0.000
T1_ODI m ± SD	37.77 ± 9.96	33.49 ± 10.27	39.50 ± 11.81	0.198
T1_TST90 m ± SD	18.20 ± 5.82	14.20 ± 4.03	24.95 ± 7.51	0.000
T1_ESS m ± SD	10.55 ± 2.08	10.15 ± 1.95	12.80 ± 2.66	0.000

**Table 3:** Comparison between group 1 and group 3.

	Perfect Diet and C-PAP Adherence N = 20	Insufficient Diet and C-PAP Adherence N = 20	*p* Value
AGE m ± SD	54.22 ± 16.88	56.37 ± 14.60	0.668
SEX n (%)	4 (20)	7 (35)	0.378
T0_BMI m ± SD	39.75 ± 3.99	38.10 ± 3.44	0.170
T0_AHI m ± SD	53.81 ± 13.99	47.02 ± 13.06	0.121
T0_ODI m ± SD	50.84 ± 15.70	43.08 ± 12.83	0.095
T0_TST90 m ± SD	28.20 ± 9.12	27.60 ± 8.24	0.828
T0_ESS m ± SD	14.85 ± 3.39	14.25 ± 2.91	0.552
T1_BMI m ± SD	32.70 ± 3.35	36.40 ± 3.21	0.001
T1_AHI m ± SD	37.68 ± 11.88	42.98 ± 11.88	0.166
T1_ODI m ± SD	37.77 ± 9.96	39.50 ± 11.81	0.620
T1_TST90 m ± SD	18.20 ± 5.82	24.95 ± 7.51	0.004
DeltaTST90	−10.00 ± 4.71	−2.65 ± 0.93	0.000
%ReductionTST90	34.56 ± 9.35	10.05 ± 3.59	0.000
T1_ESS m ± SD	10.55 ± 2.08	12.80 ± 2.66	0.005

**Table 4:** Comparison among groups of the delta parameters.

	Group 1 Perfect Diet and C-PAP Adherence Compliance N = 20	Group 2 Moderate Diet and C-PAP Adherence N = 20	Group 3 Insufficient Diet and C-PAP Adherence Compliance N = 20	*p* Value
DeltaTST90	−10.00 ± 4.71	−6.35 ± 2.43	−2.65 ± 0.93	0.000
^°,^ %ReductionTST90	34.56 ± 9.35	30.63 ± 9.78	10.05 ± 3.59	0.000
^°,^ Delta ESS	−4.30 ± 2.05	−3.80 ± 1.47	−1.45 ± 0.75	0.000
%ReductionESS	27.70 ± 11.28	27.17 ± 9.76	10.04 ± 4.87	0.000
^*,,°^ DeltaAHI m ± SD	−16.13 ± 5.18	−9.96 ± 1.78	−4.04 ± 1.62	0.000
^°,^ %ReductionAHI	30.56 ± 7.07	26.80 ± 5.64	8.58 ± 2.27	0.000
^*,,°^ DeltaBMI m ± SD	−7.05 ± 1.57	−5.54 ± 1.23	−1.70 ± 0.47	0.000
^°,^ %ReductionBMI	17.70 ± 3.37	14.98 ± 3.44	4.43 ± 1.12	0.000
^*,,°^ BMI + AHI improvement ± SD	−23.18 ± 1.18	−15.41 ± 1.82	−5.74 ± 1.82	0.000

2-group comparison. * = group 1 vs. group 2: statistically significant different parameters. ^°^ = group 1 vs. group 3: statistically significant different parameters.

**Table 5:** Intragroup comparison.

	Group 1 Perfect Diet and CPAP Adherence Compliance N = 20			Group 2 Moderate Diet and CPAP Adherence Compliance N = 20			Group 3 Insufficient Diet and CPAP Adherence Compliance N = 20		
Parameters	T0	T1	*p*	T0	T1	*p*	T0	T1	*p*
BMI m ± SD	39.75 ± 3.99	32.70 ± 3.35	0.000	36.45 ± 2.23	31.00 ± 2.44	0.000	38.10 ± 3.44	36.40 ± 3.21	0.003
AHI m ± SD	53.81 ± 13.99	37.68 ± 11.88	0.000	37.76 ± 5.72	27.80 ± 5.59	0.000	47.02 ± 13.06	42.98 ± 11.88	0.010
ODI m ± SD	50.84 ± 15.70	37.77 ± 9.96	0.000	41.19 ± 12.29	33.49 ± 10.27	0.000	43.08 ± 12.83	39.50 ± 11.81	0.003
TST 90 m ± SD	28.20 ± 9.12	18.20 ± 5.82	0.000	20.55 ± 5.16	14.20 ± 4.03	0.000	27.60 ± 8.24	24.95 ± 7.51	0.020
ESS m ± SD	14.85 ± 3.39	10.55 ± 2.08	0.000	13.95 ± 1.93	10.15 ± 1.95	0.000	14.25 ± 2.91	12.80 ± 2.66	0.020

## Data Availability

The data presented in this study are available on request from the corresponding author due to Italian privacy law.
